# Factors affecting total protein and lactoferrin in human milk

**DOI:** 10.1038/s41598-023-50124-1

**Published:** 2023-12-17

**Authors:** Bożena Kulesza-Brończyk, Agnieszka Bień, Piotr Sobieraj, Magdalena Orczyk-Pawiłowicz, Jolanta Lis-Kuberka, Matylda Czosnykowska-Łukacka, Agnieszka Bzikowska-Jura

**Affiliations:** 1 Department of Obstetrics, Gynecology and Maternity Care, Faculty of Health Sciences, Medical University of Bialystok, Białystok, Poland; 2 Chair of Obstetrics Development, Faculty of Health Sciences, Medical University of Lublin, Lublin, Poland; 3 Department of Internal Medicine, Hypertension and Vascular Diseases, Faculty of Medicine, Medical University of Warsaw, Warsaw, Poland; 4 Division of Chemistry and Immunochemistry, Department of Biochemistry and Immunochemistry, Wroclaw Medical University, Wrocław, Poland; 5 Department of Neonatology, Wroclaw Medical University, Wrocław, Poland; 6 Department of Medical Biology, Laboratory of Human Milk and Lactation Research at Regional Human Milk Bank in Holy Family Hospital, Medical University of Warsaw, Warsaw, Poland

**Keywords:** Nutrition, Public health

## Abstract

The aim of this study was to investigate factors affecting total, true protein and lactoferrin (Lf) concentrations in human milk (HM) and to evaluate the changes in protein concentrations over the course of lactation (first to sixth month postpartum). HM samples were collected from exclusively breastfeeding mothers during six time periods (1–6 months postpartum); 198 breast milk samples were collected in total. The concentrations of total and true protein in HM were determined using the MIRIS human milk analyzer (HMA). The assessment of HM protein content was also performed in skim HM samples and quantified by bicinchoninic methods with the Bicinchoninic Acid Protein Assay Kit. In turn, Lf content in skim HM samples was determined by the enzyme-linked immunosorbent assay (ELISA) in accordance with a slightly modified procedure. In the first month of lactation total protein concentration was negatively correlated with maternal pre-pregnancy BMI (r = − 0.397; *p* = 0.022), whereas in the third month postpartum, positive correlation with maternal age was found (r = 0.399; *p* = 0.021). Considering Lf concentration, in the first month of lactation, it was positively correlated with baby’s birth weight (r = 0.514; *p* = 0.002). In the next months (from second to sixth) no relationships between Lf concentration and maternal and infants’ factors were observed. The concentration of protein and Lf in HM changes dynamically throughout lactation. Maternal and infant characteristics may impact the HM protein and Lf content, especially in the first month postpartum.

## Introduction

A major factor influencing protein variability in human milk (HM) is time after delivery, with a potent decrease in protein concentration during the first month of lactation and more gradual declines in later stages^1^. HM proteins are classified into three groups: milk fat globule membrane proteins (MFGM), whey proteins and caseins. MFGM proteins constitute the smallest percentage of the true protein content in HM^2^ and their concentration is relatively stable over time^3^. The main proteins in HM are therefore caseins and whey proteins, which include α-lactalbumin, lactoferrin (Lf) and immunoglobins (mainly secretory immunoglobin A). In early lactation the concentration of whey proteins is high, whereas caseins are almost undetectable^4^. As it was reported in the metaanalysis by Lönnerdal et al.^1^, whey-casein ratio is estimated to vary from 90:10 in colostrum to 65:35 in transitional milk at then stabilizes at approximately 60:40 in mature HM.

Lf is an iron binding protein and constitutes about 20% of true protein in HM. The structure of Lf makes it resistant to proteolytic enzymes and thus, difficult to digest. It is evidenced by the presence of Lf in newborns and infants’ stool^5^. HM Lf shows anti-microbial activity against a wide range of pathogens, therefore has been demonstrated to be effective in supporting resistance to viral and bacterial infections as well as modulating immune system^6^. Evidence suggests that Lf increases not only the number but also the activity of B lymphocytes, T lymphocytes, natural killer cells, accelerates B and T cells maturation and increases the expression of cell receptors^7^.

Considering that HM is the best source of nutrition for newborns and infants, investigating the factors affecting its composition and understanding the changes over the course of lactation is a crucial first step in defining the nutritional needs of both groups. Despite many years of investigation regarding total protein and Lf concentration in HM, the results of the studies^1,8–14^ are inconsistent and, what is interesting, often are contradictory, mainly regarding to the impact of maternal and infants’ factors on HM composition. Ambiguous results may be caused by various analytical approach (e.g., lack of standardization of HM sampling, different methods for HM protein assessment) and different inclusion/exclusion criteria for mothers, so that certain results should not be compared to each other. Additionally, most of the original studies included in systemic reviews^14,15^ and metaanalysis^1^ were performed 30 years before or even more. For this reason, the comparability and sensitivity of previously used analytical methods with those currently available may be quite low. Then, one of the most important issues concerning the analysis of HM composition is standardization of expression, collection, and storage of HM samples. All these factors may affect the concentration of HM protein, and therefore the concentration of Lf also. Considered all these aspects, it seems reasonable to conduct up-to-date analysis with carefully standardized protocol.

The aim of our study was to investigate the maternal and infants’ factors affecting HM protein composition (total, true and Lf) and to evaluate the changes in proteins concentration over the course of six months of lactation in one-month interval after delivery. To increase the reproducibility and comparability of our research to other studies, the procedure regarding HM samples (expression, collection, storage, analytical methods) was highly standardized (detailed information are presented in the material and methods section). Considering that for protein analysis we used method which enable the assessment also of other macronutrients (lactose and fat)—this data was also reported.

## Material and methods

### Study design

The participants were recruited in collaboration with local midwifery service and University Hospital of Bialystok, Poland, between February and December 2021. Initially 60 breastfeeding mothers were screened for this study, however 17 did not meet the inclusion criteria (age ≥ 18 years, no chronic diseases, no smoking during or after pregnancy, sufficient milk supply, exclusive breastfeeding during the study) and in other 10 cases different situation (feeding infant with formula, need for hospitalization of the mother or infant, subsequent pregnancy, change of residence) prevented completion of the study. There were no dropouts due to maternal and/or infants’ illness (mothers and infants were healthy during the whole study period). Finally, we analyzed completed data and HM samples from 33 participants.

The study was carried out in accordance with the principles of the Declaration of Helsinki and the study protocol and ethics of this study were approved by the Bialystok Medical University Bioethics Committee (approval no. AKP.002.103.2021). All women who volunteered to participate in this study were informed about its’ purpose, provided written consent and HM samples for analysis.

### HM collection and storage

Sampling time was between 7.00 and 9.00 AM (at least an hour after the previous breastfeeding). All participants used sterilized tubes delivered by the study staff and were asked to collect about 20 mL of HM (about 10 mL of pre- and 10 mL of post-feed milk). Mothers were also instructed to wash their hands and chest area before HM expression. Collected HM samples were stored at + 4 °C until they were transported to the laboratory (it was maximum few hours, no more than five). In the laboratory, each sample was divided into two equal portions, frozen within one day and kept at − 20 °C until further analysis. To standardize the procedure of HM analysis, storage time of each HM sample before performing further analysis was between two to three months. The HM samples were obtained six times during postpartum period: 3–4 weeks (1), 7–8 weeks (2), 11–12 weeks (3), 15–16 weeks (4), 19–20 weeks (5), 23–24 weeks (6), then, in total, 198 HM samples were collected. Additionally, at first time point (3–4 weeks postpartum) data concerning maternal and infants’ characteristics was collected.

### The analysis of protein concentration in HM

Protein concentration in HM was determined using the MIRIS human milk analyzer (HMA) (Miris, Uppsala, Sweden), previously calibrated with adequate standards. The analysis was based on semisolid mid-infrared (MIR) transmission. The HMA provided a calculation of ‘total protein’ which refers to the protein content based on the total amount of nitrogen in a sample and ‘true protein’ regarding the correction for non-protein nitrogen compounds, and reflects only the content of actual protein, thus the “true” denotation. Miris HMA uses the factor 6.38 to convert N content to protein content. Additionally, HMA enabled the evaluation of fat and lactose concentrations in HM samples. The calculation of energy was based on the following conversion factors: 4.0, 9.25, 4.4 kcal per 100 mL for lactose, fat and protein, respectively.

Regarding the analysis on skim milk, HM samples were centrifuged at 3500 × g at 4 °C for 35 min, after which milk fat and cells were removed. The samples of defatted milk were stored at − 20 °C until further analysis. The concentration of protein in skim HM samples was determined with the Bicinchoninic Acid Protein Assay Kit (Sigma, St. Louis, MO, USA)^16^. For protein estimation 25 μL of 12.5- and 25-fold diluted skim milk samples and bovine albumin as a standard [from 0.2 to 1.0 mg/mL], were prepared with the use of TBS, pH = 7.5 and pipetted to the wells of microtiter plates. Afterwards, 200 μL of bicinchoninic acid working solution was added and plate was incubated at 37 °C for 35 min. In the next step, the obtained absorbance was measured in a Synergy LX Multi-Mode Reader (BioTek Instruments, Inc., Vermont, USA) at 560 nm. All skim milk samples were determined in duplicate. The intra- and inter-assay coefficients of variation were 1.0 and 2.2%, respectively.

### The analysis of Lf concentration in HM

The concentration of Lf in skim HM samples was quantified by ELISA method in accordance with a slightly modified procedure reported previously^12,17^. For determination, 100 μL of 10,000- and 20,000-fold diluted skim milk samples and a Lf standard preparation derived from human colostrum from 1.6 to 50 ng/100 μL (Sigma Aldrich, St. Louis, MO, USA) in TRIS-buffered saline (TBS, pH 7.5) were transferred to the wells of a microtiter plate (Nunc Naperville, IL, USA) and incubated at 37 °C for 2 h. For blocking and washing steps, TBS (pH 7.5) with 0.5% and 0.05% Tween-20 were used, respectively. In the next step, as a detection factor rabbit anti-human lactoferrin antibodies phosphatase-labeled (Jackson ImmunoResearch Europe Ltd., Ely, UK) in TBS with 0.05% Tween-20 were added. The enzymatic reaction was developed with a phosphatase substrate, pNPP (4-nitrophenyl phosphate), (SERVA, Heidelberg, Germany) for 15 min at 37 °C. After adding 1 M NaOH to stop enzymatic reaction, the obtained absorbance was measured at 405 nm using a Synergy LX Multi-Mode Reader. All skim milk samples were analyzed at two different dilutions, each in duplicate. The calculated intra- and inter-assay coefficients of variation were 2.1% and 7.7%, respectively.

### Statistical analysis

Continuous variables were presented as mean followed by standard deviation or median with interquartile range. Discrete variables were presented using number and percentage. Normality of the distribution was performed using Shapiro–Wilk test. Spearman or Pearson correlations coefficients were computed depending on variable distribution.

The comparison of Lf concentration measured at different time points was made using Friedman test. Post-hoc comparisons in pairs (first versus each next Lf concentration) were made using sign test with adjustment of *p*-value for multiple comparisons using Benjamini–Hochberg procedure. The results of statistical tests were considered as significant when *p*-value < 0.05.

To evaluate the relationship between Lf concentration and maternal or infant factors multivariable linear regression models were created. The best models were selected based on lowest Akaike Information Criterion (AIC) using exhaustive method (package glmulti, R 3.6.0, R Foundation for Statistical Computing, Vienna, Austria).

## Results

The mean age of participants was 30.9 ± 4.2 years. The majority of respondents had a university education (75.8%), normal pre-pregnancy weight (72.7%), the mean of weight gain during pregnancy was 15.5 ± 4.4 kg and the mean of the gestational age was 39.5 ± 1.1 weeks.

The infants’ birth weight was between 2550 and 4150 g, with a mean weight of 3392.1 ± 460.8 g. Detailed characteristic of infants’ weight gain is presented in the Table 1.

**Table 1:** Body weight characteristics of infants.

Infants’ characteristics		M ± SD	Me (Min–Max)
Body weight of the infant (g)	Birth weight (2550–4150)	3392.1 ± 460.8	3440 (2950–3800)
	Month 1	4387.0 ± 557.6	4200 (3990–4750)
	Month 2	5228.8 ± 610.0	5300 (4750–5750)
	Month 3	6048.2 ± 699.4	6100 (5430–6590)
	Month 4	6762.1 ± 670.3	6650 (6340–7200)
	Month 5	7219.7 ± 722.5	7310 (6790–7580)
	Month 6	7687.3 ± 809.6	7740 (7200–8400)
Infants’ weight gain (g)^1^	Month 1	994.8 ± 360.6	930 (790–1250)
	Month 2	841.8 ± 318.4	790 (680–950)
	Month 3	819.4 ± 362.5	800 (610–970)
	Month 4	713.9 ± 338.3	770 (460–900)
	Month 5	457.6 ± 207.0	450 (310–520)
	Month 6	467.6 ± 189.4	460 (360–520)

*M* mean, *SD* standard deviation, *Me* median, ^1^infants’ weight gain during each month was calculated as the difference between current weight and weight in the previous month.

**Figure 1:**
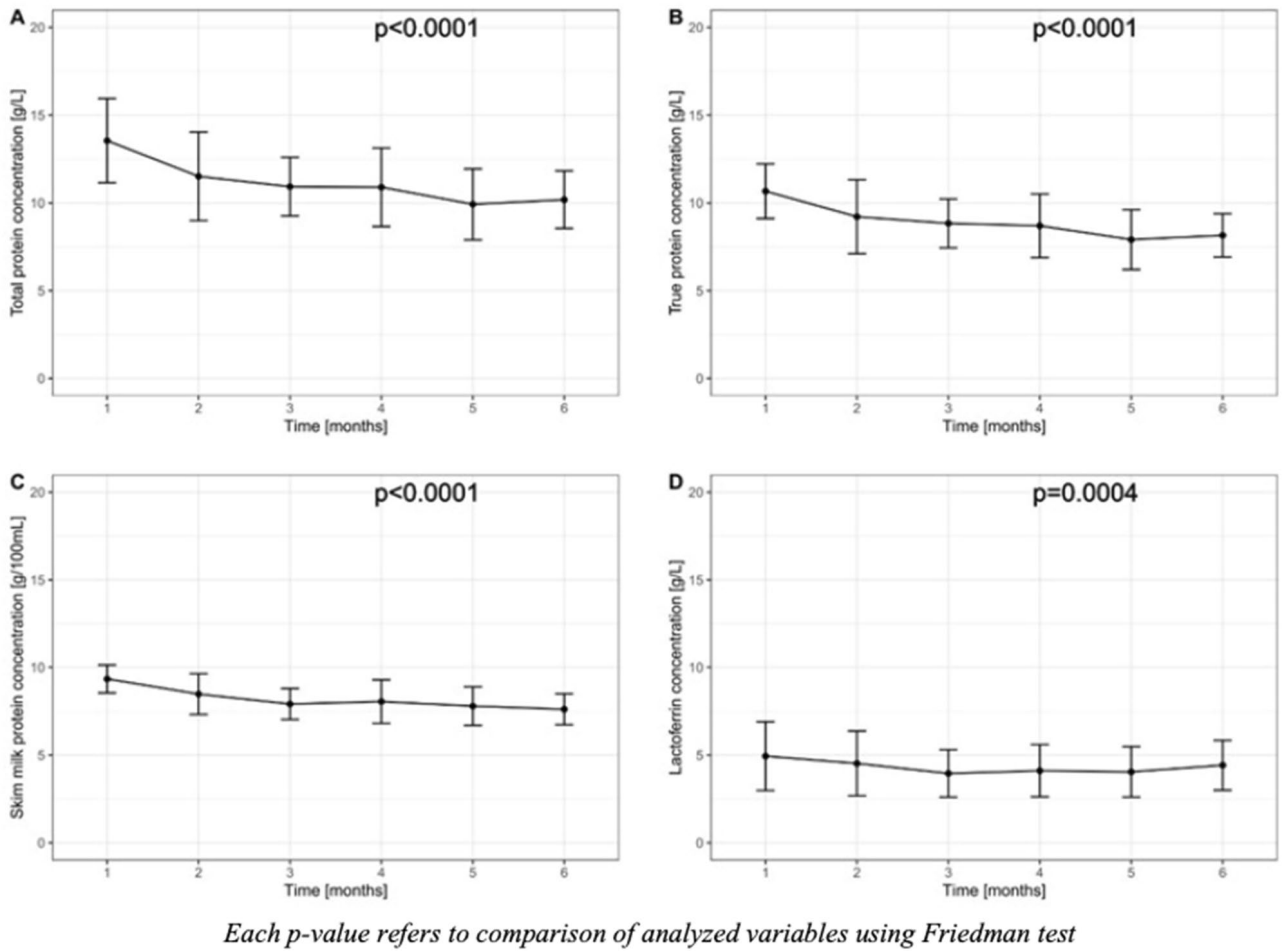
Concentration of total protein (panel **A**), true protein (panel **B**), skim milk protein concentration (Panel **C**) and lactoferrin (Panel **D**) during the study period.

The concentration of total protein (*p* < 0.0001), true protein (*p* < 0.0001), skim milk protein (*p* < 0.0001) and Lf concentration (*p* = 0.0004) changed over the time during the six-month observation. Total and true protein content in HM tended to decrease over the first five months postpartum and then increased in the sixth month, while protein concentration in skim milk decreased gradually until third month, increased in month four, and then continue to decrease in the subsequent months (Fig. 1).

Additionally, in the Table 2 we presented data concerning fat, lactose, dry mass, and energy content in HM samples over the six months postpartum. No significant differences were found for both nutrients and energy value at different lactation stages (*p* > 0.05).

**Table 2:** The concentration of other macronutrients and energy in human milk samples over the six months of lactation.

Component	Month 1 M ± SD	Month 2 M ± SD	Month 3 M ± SD	Month 4 M ± SD	Month 5 M ± SD	Month 6 M ± SD	*p*-value
Fat (g/100 mL)	3.35 ± 1.32	3.10 ± 1.25	3.09 ± 1.51	3.11 ± 1.47	3.19 ± 1.52	3.68 ± 1.68	0.551
Lactose (g/100 mL)	7.80 ± 0.39	7.80 ± 0.36	7.87 (0.34)	7.72 ± 0.98	8.02 ± 0.71	7.94 ± 0.29	0.324
Energy (kcal/100 mL)	68.45 ± 12.09	64.03 ± 10.12	64.52 (13.69)	63.50 ± 14.71	64.71 ± 13.70	71.06 ± 14.93	0.137
Total dry matter (g/100 mL)	12.68 ± 1.34	12.22 ± 1.25	12.17 (1.49)	11.99 ± 1.87	12.07 ± 1.57	12.63 ± 1.71	0.333

*M* mean, *SD* standard deviation.

In the first month postpartum total protein concentration was negatively correlated with maternal pre-pregnancy BMI (r = − 0.397, *p* = 0.022). Additionally, maternal age was positively correlated with total protein content in the third month postpartum (r = 0.399; *p* = 0.021). In the first month of lactation, Lf concentration was positively correlated with baby’s birth weight (r = 0.514; *p* = 0.002). In the following months no relationships between Lf concentration and maternal and infants’ factors were observed. Detailed information about correlations between total protein and Lf content in HM were presented in the Table 3.

**Table 3:** Correlations between maternal and infant factors and total protein and lactoferrin concentrations in HM in the 1st, 2nd, and 3rd months postpartum.

	Total protein			Lactoferrin		
	1	3	6	1	3	6
Maternal age (years)	− 0.094	0.399*	0.045	− 0.242	− 0.146	− 0.080
Pre-pregnancy BMI (kg/m^2^)	− 0.397*	− 0.019	− 0.059	− 0.017	− 0.206	− 0.311
Weight gain during pregnancy (kg)	− 0.023	− 0.141	0.172	0.255	0.299	0.270
Infant’s birth weight (g)	0.019	− 0.029	− 0.101	0.514*	0.314	0.331
Infant’s weight gain^1^ (g)	− 0.207	− 0.310	− 0.347	− 0.400	− 0.097	− 0.287
Infant’s current weight (g)	− 0.099	− 0.224	− 0.308	0.227	0.295	− 0.041

Pearson’s correlation coefficient or Spearman’s correlation coefficient were used, depending on variable distribution. ^1^Infant’s weight gain was calculated as the difference between the current body weight (in each month) and body weight in the previous month. *p < 0.05.

Considering fat, lactose, and energy content in HM samples, they were usually not associated with maternal and infants’ factors (Table 4). The only significant correlation that we have found was for energy value in the first month postpartum and pre-pregnancy BMI (r = 0.218, *p* < 0.05).

**Table 4:** Correlations between maternal age, pre-pregnancy BMI, infants’ weight and HM fat, lactose, and energy content.

	Fat			Lactose			Energy		
	1	3	6	1	3	6	1	3	6
Age (years)	− 0.046	− 0.251	0.111	− 0.066	− 0.030	0.108	− 0.070	− 0.235	0.138
Pre-pregnancy BMI (kg/m^2^)	0.232	− 0.236	− 0.159	0.201	− 0.076	0.33	0.218*	− 0.257	− 0.203
Infant’s current weight (g)	0.226	0.216	0.137	0.003	− 0.142	− 0.047	0.169	0.202	0.197

^1^Pearson’s correlation coefficient or Spearman’s correlation coefficient were used, depending on variable distribution. ^2^Infant’s weight gain was calculated as the difference between the current body weight (in each month) and body weight in the previous month. *p < 0.05.

Among considered linear regression models, Lf concentration in the first month was dependent on birth weight (estimate 0.002, *p* = 0.007) and infant weight gain (estimate − 0.002, *p* = 0.032). Lf concentration in the third month was dependent on pre-pregnancy BMI (estimate − 0.153, *p* = 0.055) and total pregnancy weight gain (estimate 0.115, *p* = 0.032). Lf concentration in the sixth month was dependent on pre-pregnancy BMI (− 0.172, *p* = 0.038), total pregnancy weight gain (estimate 0.088, *p* = 0.120) and birth weight (estimate 0.001, *p* = 0.171). Detailed models are presented in Table 5.

**Table 5:** Models evaluating relationship between maternal or infant factors and lactoferrin concentration in the 1st (Model A), 3rd (Model B) and 6th month (Model C).

Model A—prediction of Lf concentration in the 1st month		
Variable	Estimate	*p*-value
Intercept	0.427	0.860
Birth weight (g)	0.002	0.007
Infant’s weight gain (g)	− 0.002	0.032
Model B—prediction of Lf concentration in the 3rd month		
Variable	Estimate	*p*-value
Intercept	5.503	0.003
Pre-pregnancy BMI (kg/m2)	− 0.153	0.055
Pregnancy weight gain (kg)	0.115	0.032
Model C—prediction of Lf concentration in the 6th month		
Variable	Estimate	*p*-value
Intercept	4.335	0.085
Pre-pregnancy BMI (kg/m2)	− 0.172	0.038
Pregnancy weight gain (kg)	0.088	0.120
Birth weight (g)	0.001	0.171

*Lf* lactoferrin, *BMI* Body Mass Index.

## Discussion

In the present study, we found that the concentrations of protein and Lf in HM changed significantly over the first six month of lactation. Considering both, higher values were observed in the first month postpartum. Maternal anthropometric factors (pre-pregnancy BMI and weight gain during pregnancy) affected Lf content in HM, and additionally, we reported positive correlation with baby’s birth weight. For total protein, in the first month, negative correlation with maternal pre-pregnancy BMI was observed.

The mean true protein concentration in our HM samples was 1.07 ± 0.16 g/100 mL in the first month and then gradually decreased. These results are consistent with reports of other authors^8,9^ and reflect the adaptation of HM composition to the increased energy demand of infants during a period of rapid growth. In their metaanalysis, Lönnerdal et al.^1^, performed linear regression analysis to characterize the dynamic evolution of true protein concentration over time. Considering methodology of this analysis it is worth to mention that only selected studies included in the metaanalysis provided sufficient information regarding geographic location, study design, sampling time and procedure, analytical methods, and units. All these factors may impact HM protein concentration. Nonetheless, the authors reported that by 90–360 days, true protein content in HM was 47% lower compared to 0 to 5 days postpartum. Similar results were obtained by Zhang et al.^9^, who demonstrated that protein concentration decreased over six months after delivery from 1.67 mg/100 mL (one to five days postpartum) to 0.99 mg/100 mL in the sixth month of lactation (*p* < 0.001). Additionally, the authors^9^ reported that several maternal factors impacted the longitudinal changes in HM proteins concentrations. For example, they have found that women aged ≥ 30 years had a relatively lower concentration of total protein in the colostrum but a higher concentration in mature HM. In our study we found positive correlation with maternal age in the third month postpartum. Secondly, Zhang et al.^9^ observed that higher maternal education was related to a higher concentration of total protein in early HM, higher α-lactalbumin in colostrum, and higher osteopontin in mature milk. They explained it by the different nutritional habits which depends on maternal education and income. In our study, instead of age, the only maternal factors affecting HM protein concentration was maternal pre-pregnancy BMI (negative correlation in the first month postpartum), similarly to Quinn et al.^10^. However, it is worth to mention that BMI is not a direct measure of adiposity, so that the strength of this relationship may not reflect the true value of these associations. Especially since, the results of many studies^18–22^ have shown positive relationship between maternal fat mas (%) and HM protein concentration. Therefore, the results of our study may indicate that the BMI values in the study group was determined by the high content of muscle mass and not fat mass. Additionally, interesting results in this case were obtained by Bachour et al.^23^ who reported that HM protein content was significantly negatively associated with maternal BMI—normal vs overweight: 1.52 ± 0.07 vs 1.29 ± 0.06 mg/100 mL (*p* = 0.044) but not for normal vs obese (1.52 ± 0.07 vs 1.59 ± 0.02).

Rai et al.^14^ performed a global systemic review which aimed to rigorously consider all available studies (n = 94) providing data about HM Lf and factors which affect its concentration. The authors concluded that Lf concentration was the highest during early lactation and rapidly declined to remain relatively stable from one month to next months postpartum. The mean of the means (± SEM) of Lf concentration in early milk (< 28 days) was 4.91 ± 0.31 g/L and 2.1 ± 0.87 g/100 L in mature milk. One of the basic limitations of this review was a fact that most of the included studies was carried out in the eighties, nineties or even before. Thus, the methodology used may affect the ability to compare these results with current data. What is more, as ‘early milk’ the authors defined all samples < 28 days of lactation, indicating while the highest Lf concentration is in colostrum (the first 5 days of life) − 6.63 ± 3.74, decreases to 50% by days 6–10, and plateaus out after the first month of life. Villavicencio et al.^13^ also underlined that Lf concentration in colostrum is the highest. Therefore, mean values concerning all data from the first month (< 28 days) should be interpreted with caution. Regarding Lf concentration in HM, we observed significant differences in its concentration over the course of lactation (*p* = 0.0004), which was similar to results obtained by Affolter et al.^11^, who reported following values 3.30 g/L at 5–11 days and 1.17 g/L at 4–8 months postpartum. Another study^20^ showed that the Lf content in HM was higher in samples from mother who breastfed > 12 months (5.0 g/L) in comparison to mothers who breastfed between 1 and 12 months (3.4 g/L). This indicates that milk after one year of lactation has a high immunomodulatory potential, comparable to that of colostrum. In our study we observed that Lf concentration in the first month postpartum was significantly different than in the next five months. Affolter et al.^11^ obtained consistent results and observed significant differences in Lf concentration between the first three investigated stages (5–11 days, 12–30 days, and 1–2 months) This variability in the Lf content may reflect the different needs of different infants during the early weeks of life. Additionally, this temporal pattern of change in Lf concentration in HM (between first and next months) parallels that observed for total protein content through postpartum period. It also must be stressed that in our study the collection of HM samples was standardized (morning sampling time, pre- and post-feed samples), whereas in other studies^24–26^ the procedure of milk expression was not specified which may impact HM protein content and thus, also Lf concentration. Studies which aimed to analyze the effect of various factors (geographical location, maternal socio-demographic data, perinatal parameters) on Lf level in HM, mainly found no significant impacts^13,14^. In our study, Lf concentration in HM was dependent on maternal pre-pregnancy BMI, total weight gain during pregnancy and baby’s birth weight (Table 3). In turn, Albenzio et al.^27^ reported the highest Lf level in HM samples of mothers of extremely pre-mature newborns compared with those with higher gestational age and term pregnancy (*p* < 0.001). For the present analysis we included only full-term pregnancy mothers, however, in the further analysis, we plan to involve mother who delivered prematurely, especially since in the systemic review of Rai et al.^14^, the authors underlined that there is limited number of data concerning Lf concentration in preterm milk.

Our study was performed according to standardized methods (including HM collection and HM analysis); however, some crucial limitations must be underlined. Firstly, considering the preliminary character of this publication, at this stage, the number of participated women is modest. Secondly, most of the mothers had normal weight, higher education and lived in urban areas which affect the representativity of the study population. Thirdly, the morning hours of HM collection may impact protein and Lf concentration in HM, so the caution should be used when comparing the results from other studies (when e.g. 24-h collection procedure was used). Finally, the storage time of HM samples before performing the analysis of Lf concentration may slightly differ between samples (maximum 4 weeks). As it was reported by other authors^28,29^, Lf concentration decreases after three and six months at − 20 °C. It was suggested that the largest loss of Lf occurs with the initial act of freezing. Nonetheless, some differences in Lf concentration in our HM samples may be due to small differences in storage time in freezing conditions.

## Conclusions

To conclude, the concentration of protein (total, true, skim milk protein) and Lf in HM changes dynamically throughout lactation. Maternal and infant characteristics may impact the HM protein and Lf content, especially in the first month postpartum.

## Data Availability

The datasets used and analysed during the current study available from the corresponding author on reasonable request.
